# Supplementary material for: Effect of the Number of Anchoring and Electron-Donating Groups on the Efficiency of Free-Base- and Zn-Porphyrin-Sensitized Solar Cells
Source: Materials (Basel). 2019 Feb 21;12(4):650. doi: 10.3390/ma12040650 (PMC6416604; doi:10.3390/ma12040650)
Supplement: Supplementary file 1 [file materials-12-00650-s001.pdf]

materials

Supporting Information: Effect of the Number of Anchoring and Electron Donating Groups on the efficiency of Free-Base- and Zn-Porphyrin-Sensitized Solar Cells

Raheleh Nasrollahi [1,2], Luis Martín-Gomis [1], Fernando Fernández-Lázaro [1], Saeed Zakavi [2] and Ángela Sastre-Santos [1,*]

Figure S1. Molecular structure of H_2P-CO_2H 1. 3
Figure S2. ^1H-NMR ($CDCl_3$) H_2P-CO_2H 1. 3
Figure S3. HR-MS (MALDI-TOF) spectrum of H_2P-CO_2H 1. 3
Figure S4. Molecular structure of H_2P-$(CO_2H)_2$ 2 cis. 4
Figure S5. ^1H-NMR ($CDCl_3$) of H_2P-$(CO_2H)_2$ 2 cis. 4
Figure S6. HR-MS (MALDI-TOF) spectrum of H_2P-$(CO_2H)_2$ 2 cis. 4
Figure S7. Molecular structure of H_2P-$(CO_2H)_2$ 2 trans. 5
Figure S8. ^1H-NMR ($CDCl_3$) of H_2P-$(CO_2H)_2$ 2 trans. 5
Figure S9. HR-MS (MALDI-TOF) spectrum of H_2P-$(CO_2H)_2$ 2 trans. 5
Figure S10. Molecular structure of H_2P-$(CO_2H)_3$ 3. 6
Figure S11. ^1H-NMR ($CDCl_3$) of H_2P-$(CO_2H)_3$ 3. 6
Figure S12. HR-MS (MALDI-TOF) spectrum of H_2P-$(CO_2H)_3$ 3. 6
Figure S13. Molecular structure of ZnP-CO_2H 4. 7
Figure S14. ^1H-NMR ($CDCl_3$) of ZnP-CO_2H 4. 7
Figure S15. HR-MS (MALDI-TOF) spectrum of ZnP-CO_2H 4. 7
Figure S16. Molecular structure of ZnP-$(CO_2H)_2$ 5 cis. 8
Figure S17. ^1H-NMR ($CDCl_3$) of ZnP-$(CO_2H)_2$ 5 cis. 8
Figure S18. HR-MS (MALDI-TOF) spectrum of ZnP-$(CO_2H)_2$ 5 cis. 8
Figure S19. Molecular structure of ZnP-$(CO_2H)_2$ 5 trans. 9
Figure S20. ^1H-NMR ($CDCl_3$) of ZnP-$(CO_2H)_2$ 5 trans. 9
Figure S21. HR-MS (MALDI-TOF) spectrum of of ZnP-$(CO_2H)_2$ 5 trans. 9
Figure S22. Molecular structure of ZnP-$(CO_2H)_3$ 6. 10
Figure S23. ^1H-NMR ($CDCl_3$) of ZnP-$(CO_2H)_3$ 6. 10
Figure S24. HR-MS (MALDI-TOF) spectrum of of ZnP-$(CO_2H)_3$ 6. 10
Figure S25. Molecular structure of H_2P-CO_2Me 7. 11
Figure S26. ^1H-NMR ($CDCl_3$) of H_2P-CO_2Me 7. 11
Figure S27. HR-MS (MALDI-TOF) spectrum of H_2P-CO_2Me 7. 11
Figure S28. Molecular structure of H_2P-$(CO_2Me)_2$ 8, mixture of isomers. 12
Figure S29. ^1H-NMR ($CDCl_3$) of H_2P-$(CO_2Me)_2$ 8, mixture of isomers. 12
Figure S30. HR-MS (MALDI-TOF) spectrum of H_2P-$(CO_2Me)_2$ 8, mixture of isomers. 12
Figure S31. Molecular structure of H_2P-$(CO_2Me)_3$ 9. 13
Figure S32. ^1H-NMR ($CDCl_3$) of H_2P-$(CO_2Me)_3$ 9. 13
Figure S33. HR-MS (MALDI-TOF) spectrum of H_2P-$(CO_2Me)_3$ 9. 13

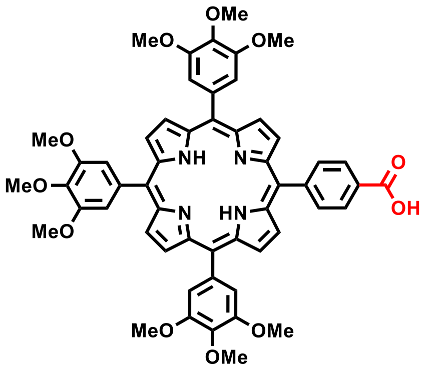

Figure S1. Molecular structure of H2P-CO2H 1.

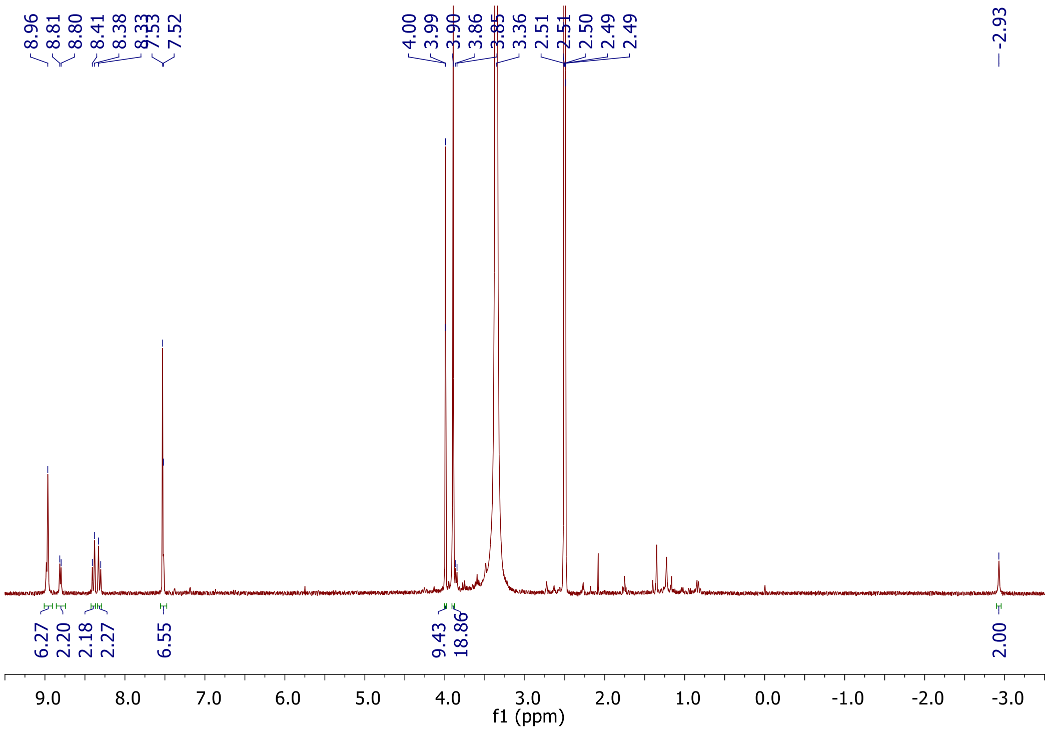

Figure S2. ^{1}H-NMR (CDCl3) H2P-CO2H 1.

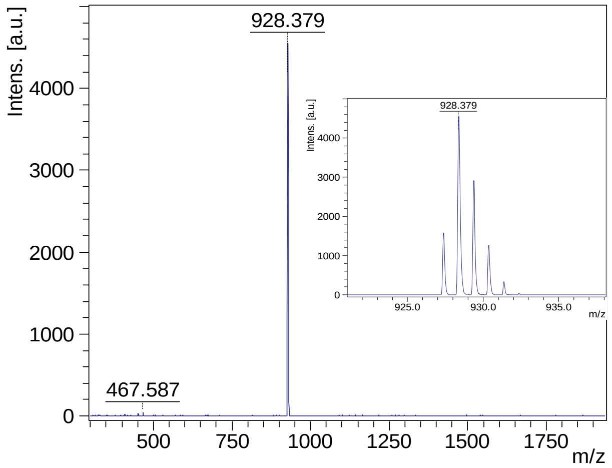

Figure S3. HR-MS (MALDI-TOF) spectrum of H2P-CO2H 1.

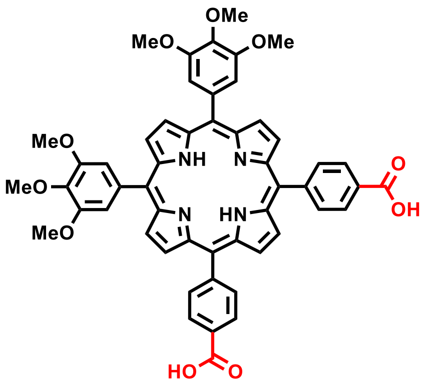

Figure S4. Molecular structure of $H_2P\text{-}(CO_2H)_2$ 2 cis.

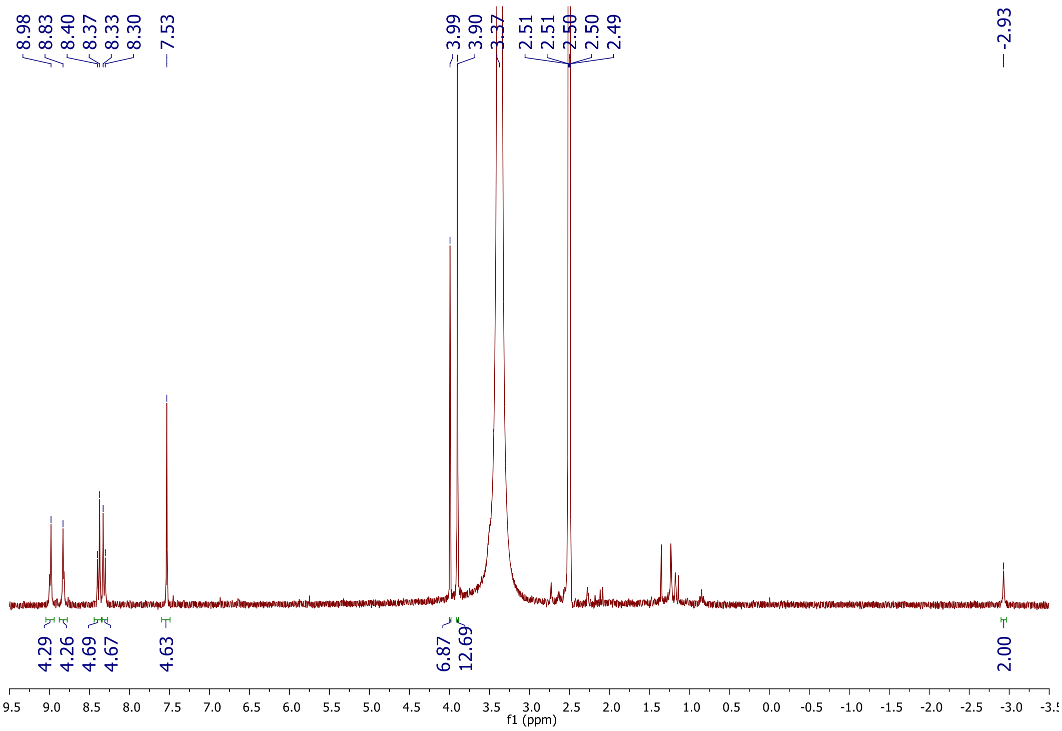

Figure S5. ^{1}H-NMR ($CDCl_3$) of $H_2P\text{-}(CO_2H)_2$ 2 cis.

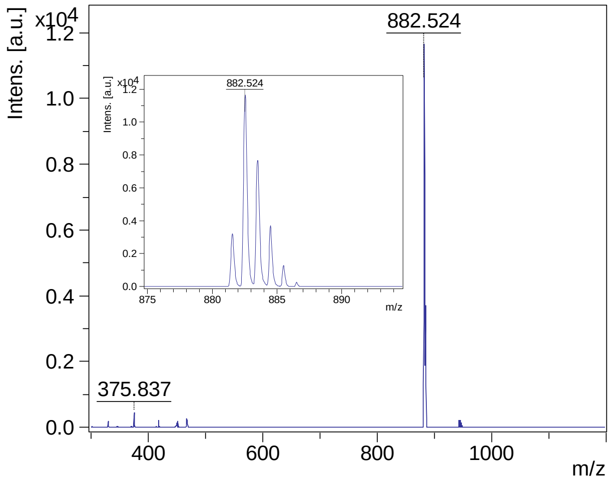

Figure S6. HR-MS (MALDI-TOF) spectrum of $H_2P\text{-}(CO_2H)_2$ 2 cis.

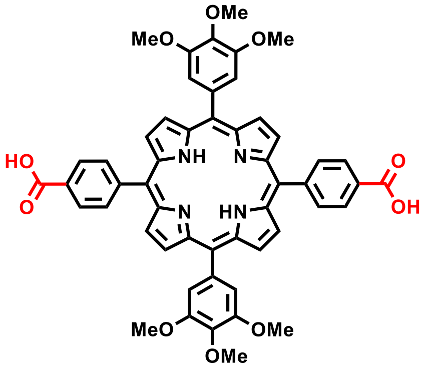

Figure S7. Molecular structure of H_2P-$(CO_2H)_2$ 2 trans.

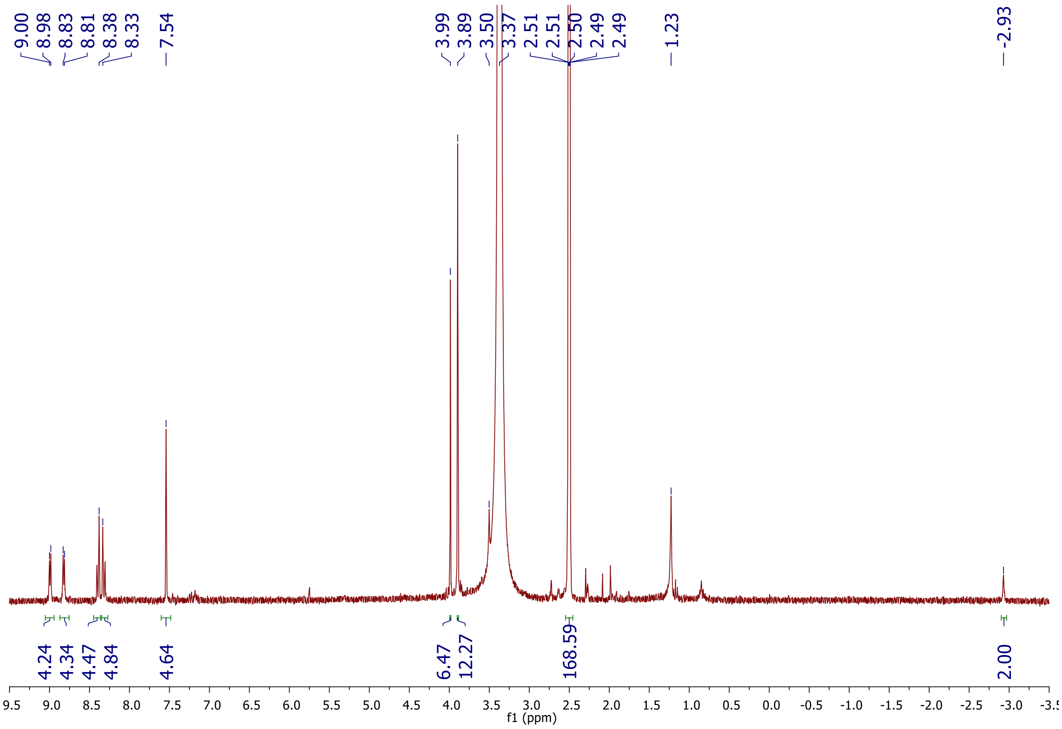

Figure S8. ^{1}H-NMR ($CDCl_3$) of H_2P-$(CO_2H)_2$ 2 trans.

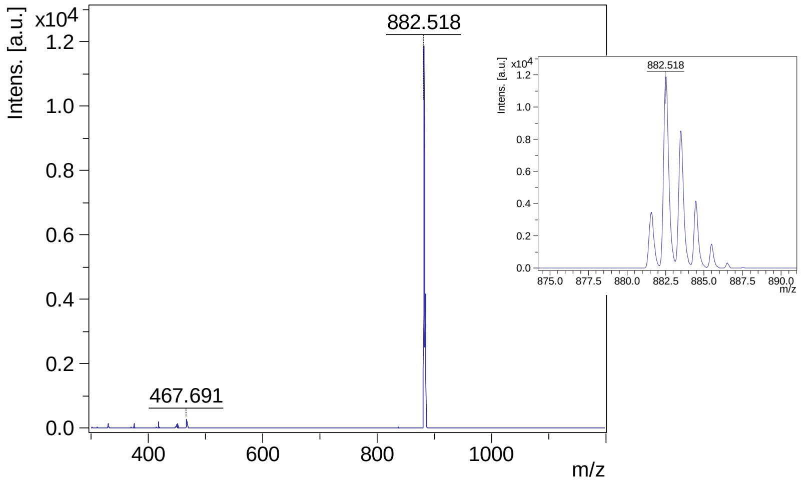

Figure S9. HR-MS (MALDI-TOF) spectrum of H_2P-$(CO_2H)_2$ 2 trans.

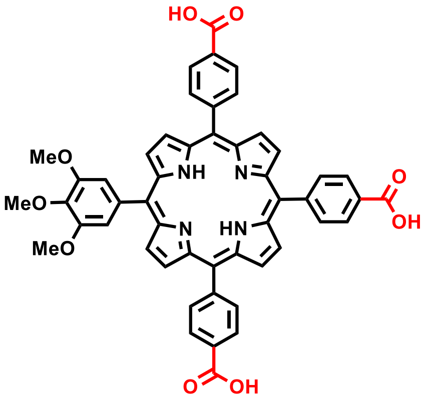

Figure S10. Molecular structure of H_2P-$(CO_2H)_3$ 3.

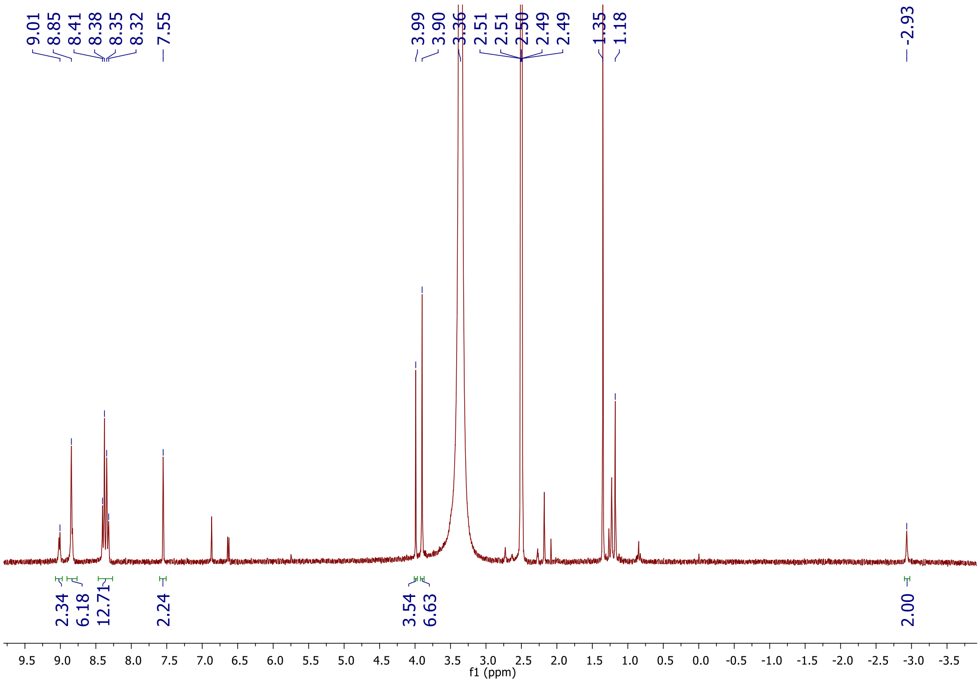

Figure S11. ¹H-NMR ($CDCl_3$) of H_2P-$(CO_2H)_3$ 3.

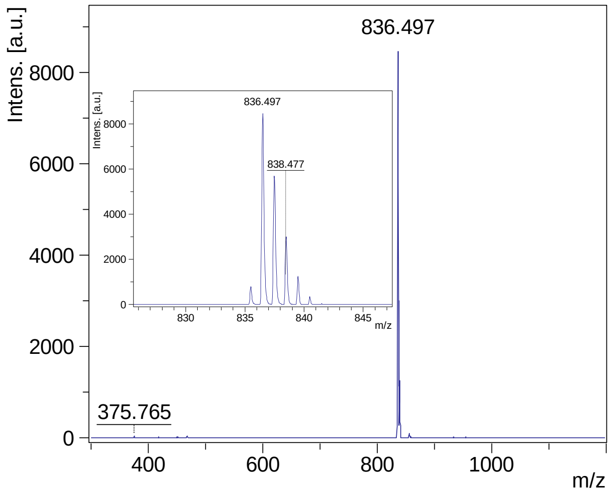

Figure S12. HR-MS (MALDI-TOF) spectrum of H_2P-$(CO_2H)_3$ 3.

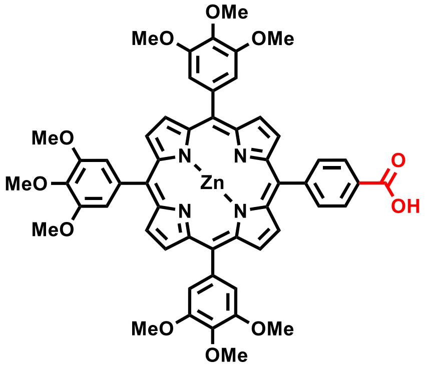

Figure S13. Molecular structure of ZnP-CO_2H 4.

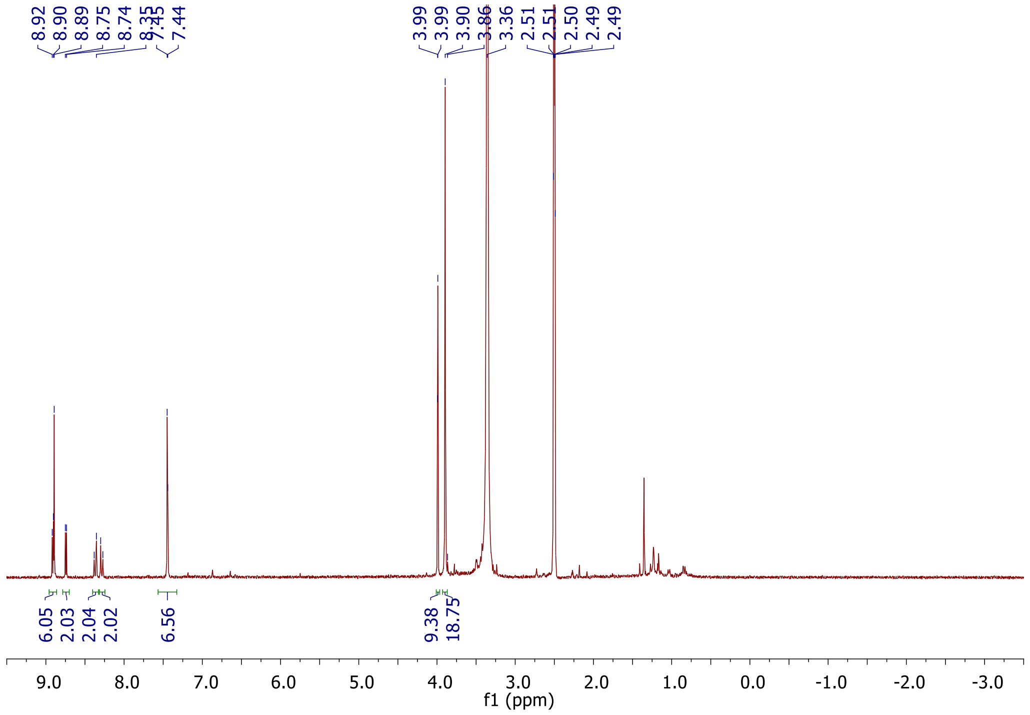

Figure S14. ^{1}H-NMR ($CDCl_3$) of ZnP-CO_2H 4.

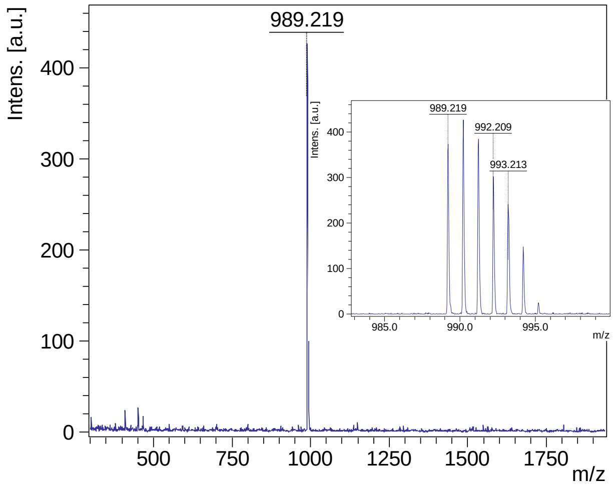

Figure S15. HR-MS (MALDI-TOF) spectrum of ZnP-CO_2H 4.

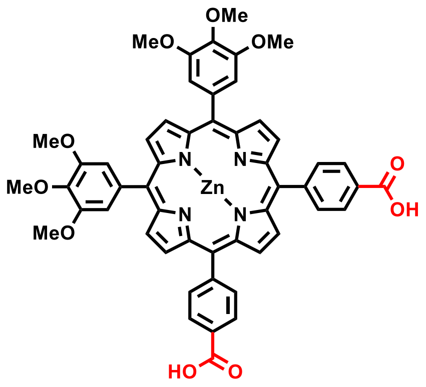

Figure S16. Molecular structure of ZnP-$(CO_2H)_2$ 5 cis.

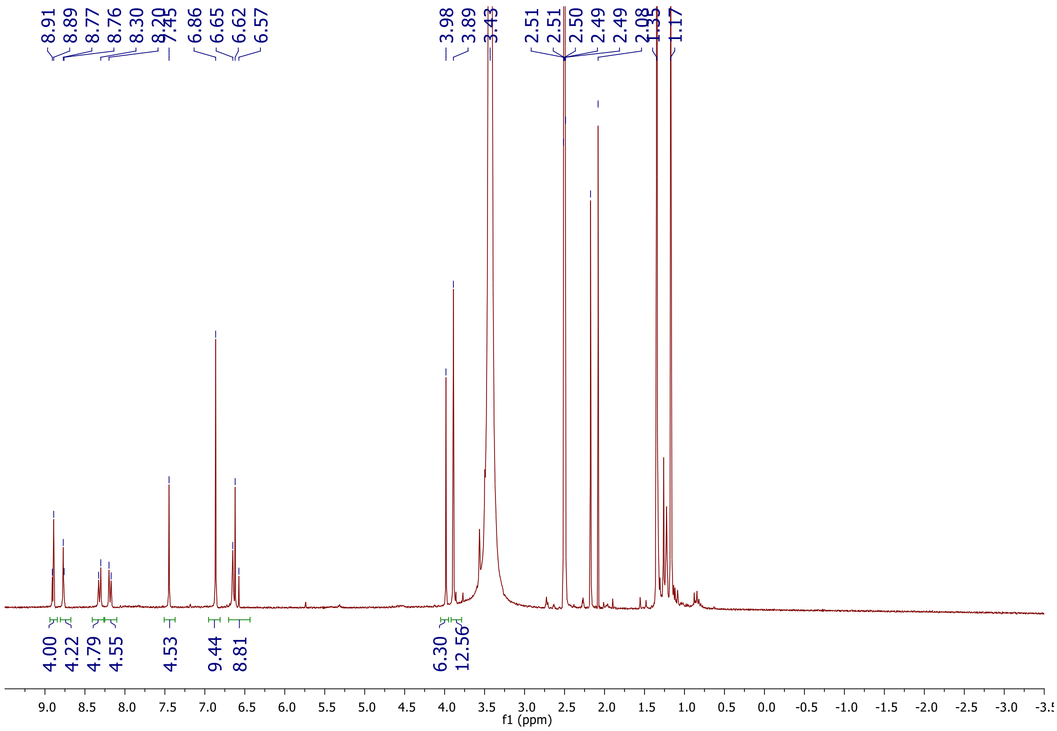

Figure S17. ^{1}H-NMR ($CDCl_3$) of ZnP-$(CO_2H)_2$ 5 cis.

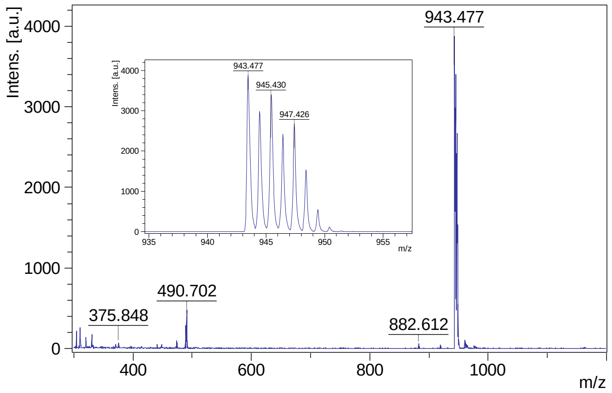

Figure S18. HR-MS (MALDI-TOF) spectrum of ZnP-$(CO_2H)_2$ 5 cis.

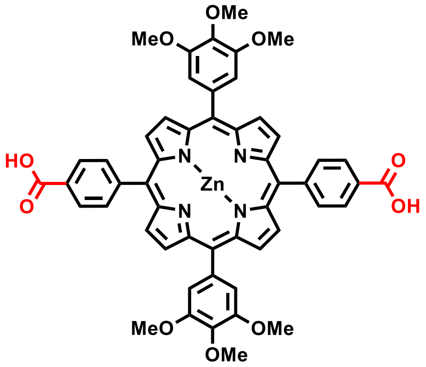

Figure S19. Molecular structure of ZnP-$(CO_2H)_2$ 5 trans.

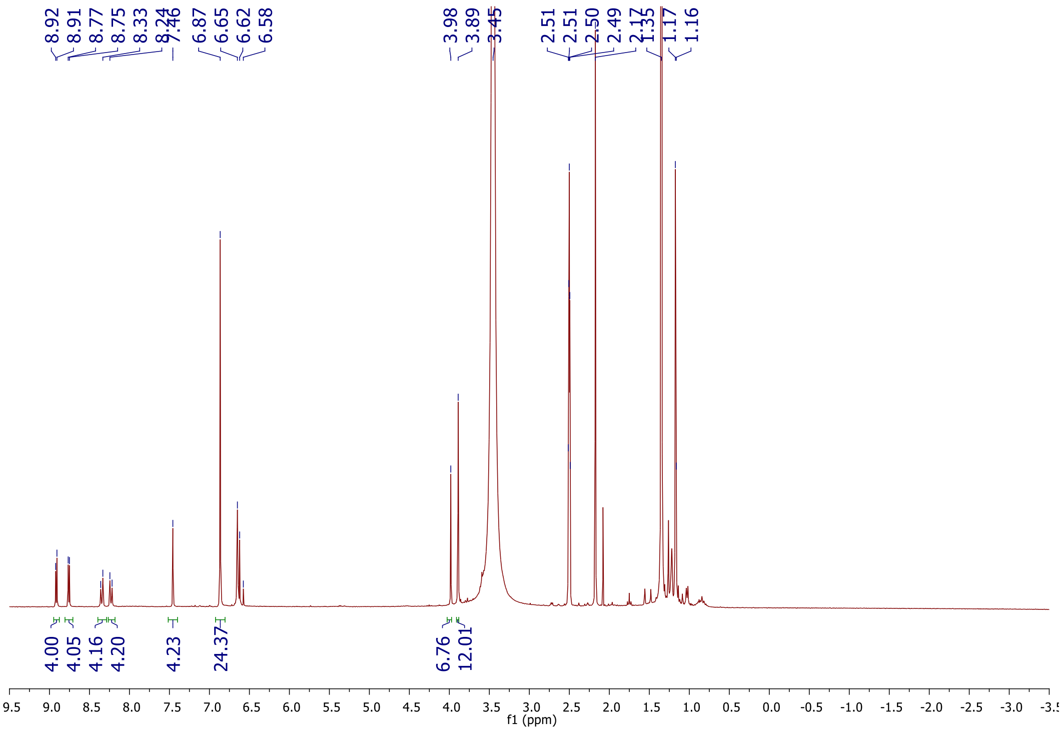

Figure S20. ^{1}H-NMR ($CDCl_3$) of ZnP-$(CO_2H)_2$ 5 trans.

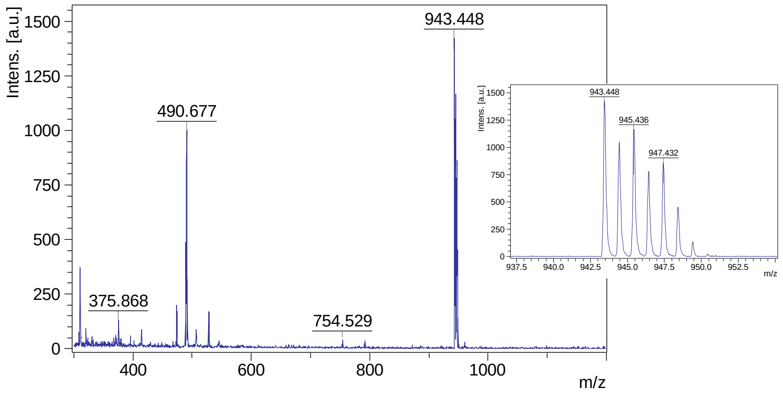

Figure S21. HR-MS (MALDI-TOF) spectrum of of ZnP-$(CO_2H)_2$ 5 trans.

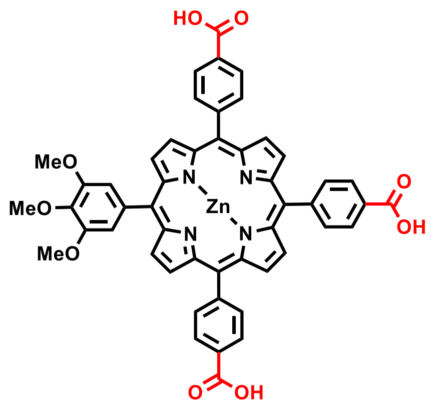

Figure S22. Molecular structure of ZnP-$(CO_2H)_3$ 6.

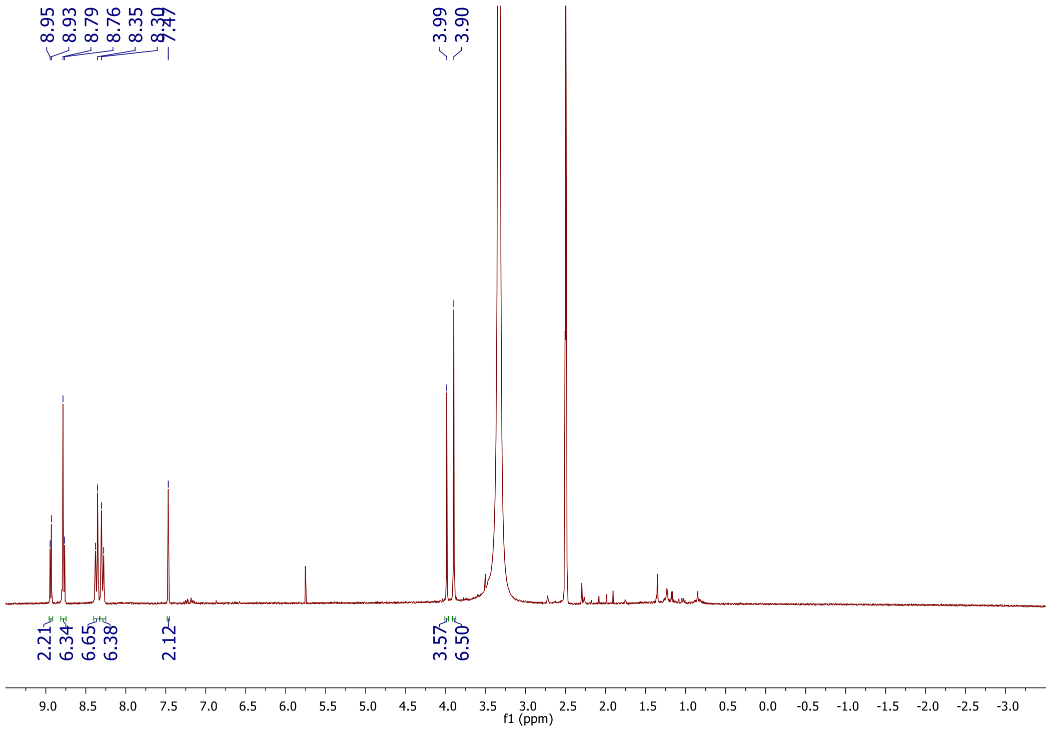

Figure S23. ^{1}H-NMR ($CDCl_3$) of ZnP-$(CO_2H)_3$ 6.

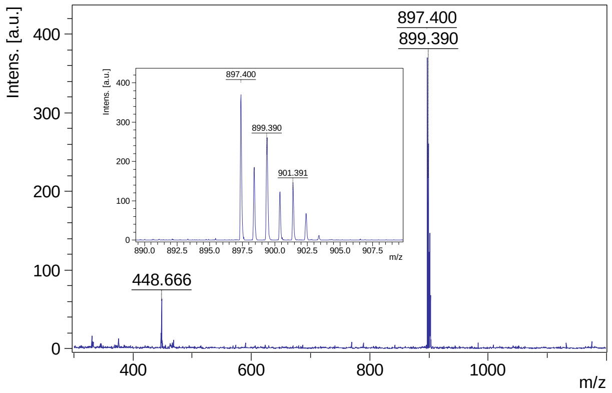

Figure S24. HR-MS (MALDI-TOF) spectrum of of ZnP-$(CO_2H)_3$ 6.

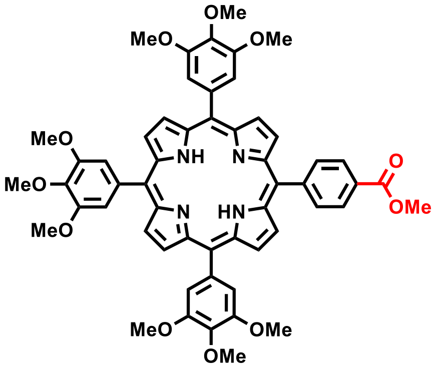

Figure S25. Molecular structure of $H_2P\text{-}CO_2Me$ 7.

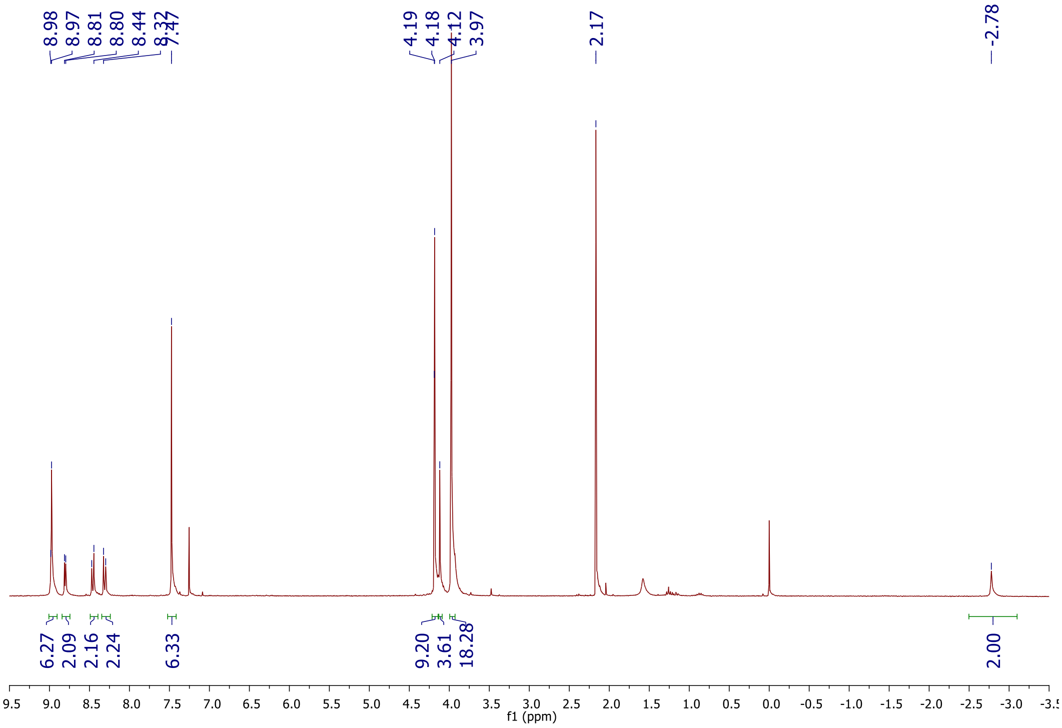

Figure S26. ^{1}H-NMR ($CDCl_3$) of $H_2P\text{-}CO_2Me$ 7.

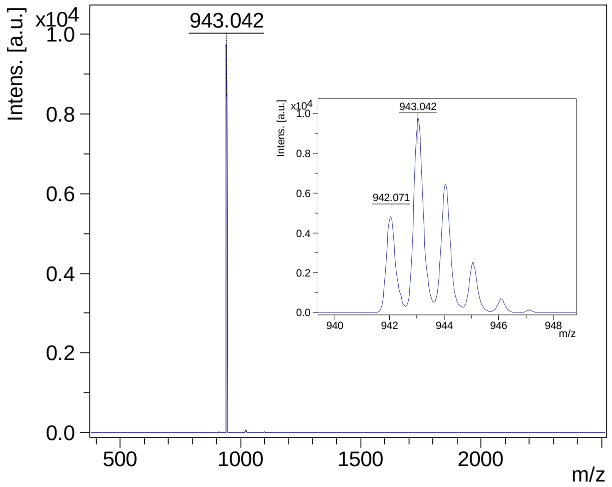

Figure S27. HR-MS (MALDI-TOF) spectrum of $H_2P\text{-}CO_2Me$ 7.

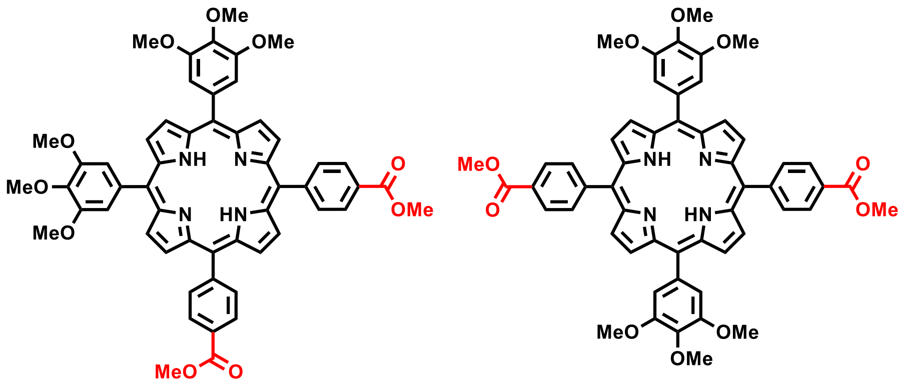

Figure S28. Molecular structure of H_2P-$(CO_2Me)_2$ 8, mixture of isomers.

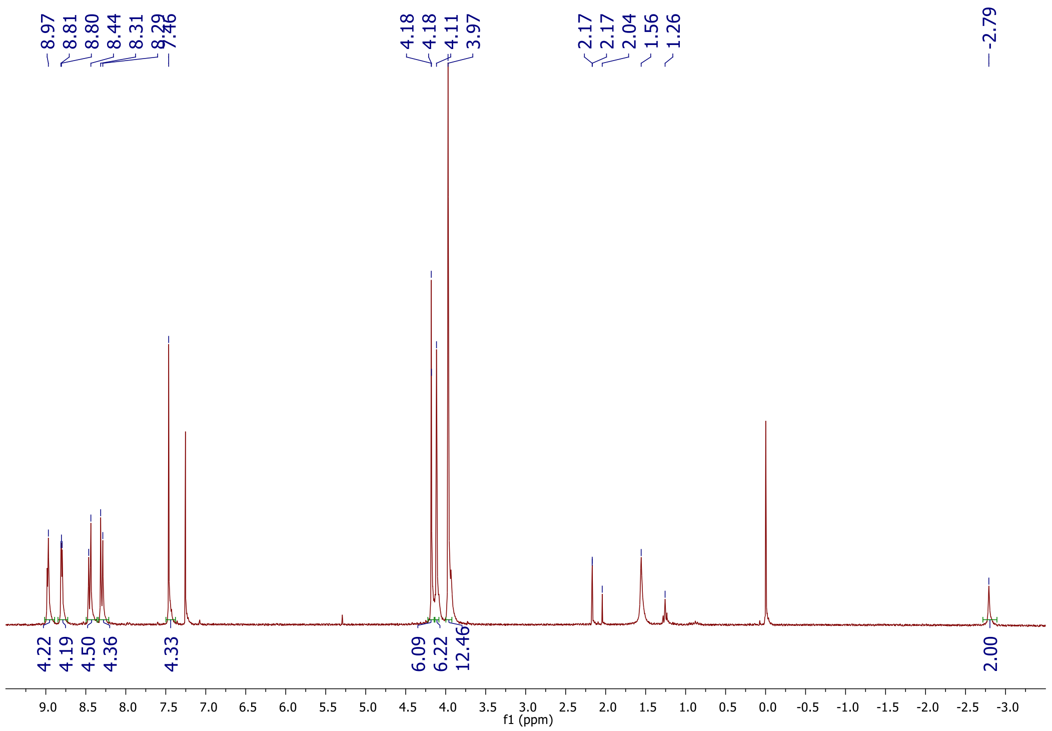

Figure S29. ^{1}H-NMR ($CDCl_3$) of H_2P-$(CO_2Me)_2$ 8, mixture of isomers.

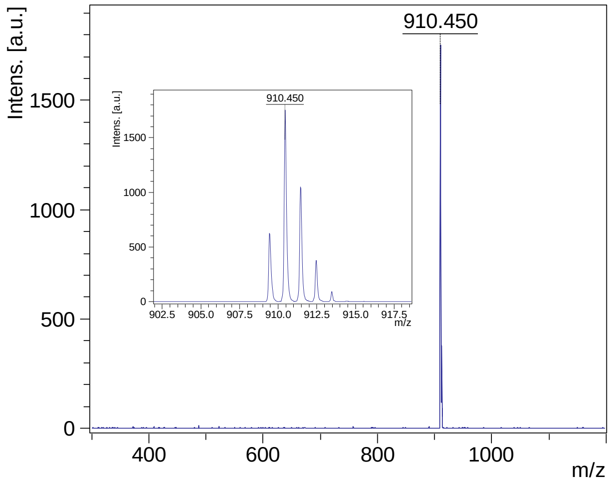

Figure S30. HR-MS (MALDI-TOF) spectrum of H_2P-$(CO_2Me)_2$ 8, mixture of isomers.

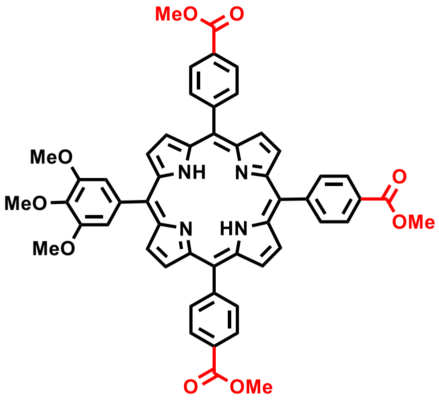

Figure S31. Molecular structure of H_2P-$(CO_2Me)_3$ 9.

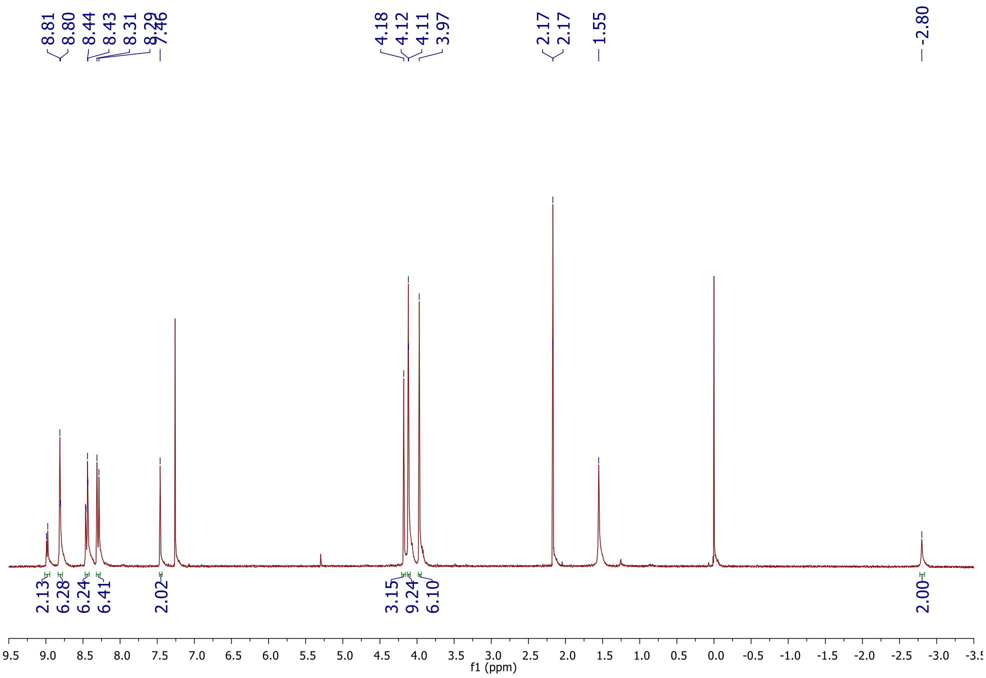

Figure S32. ^{1}H-NMR ($CDCl_3$) of H_2P-$(CO_2Me)_3$ 9.

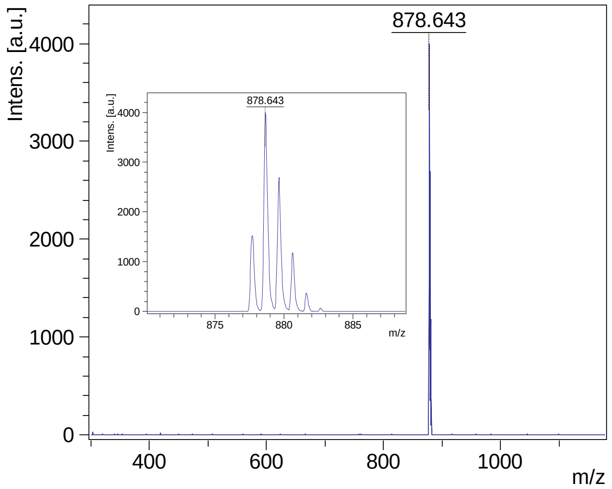

Figure S33. HR-MS (MALDI-TOF) spectrum of H_2P-$(CO_2Me)_3$ 9.

© 2018 by the authors. Submitted for possible open access publication under the terms and conditions of the Creative Commons Attribution (CC BY) license (http://creativecommons.org/licenses/by/4.0/).
